# Aesthetics of Numerical Proportions in Human Cosmetic Surgery

**DOI:** 10.29252/wjps.8.1.78

**Published:** 2019-01

**Authors:** Zhaleh Shahbazi, Hossein Ardalani, Mahsa Maleki

**Affiliations:** Department of Art and Architecture, Hamedan Branch, Islamic Azad University, Hamedan, Iran

## Abstract

**BACKGROUND:**

Beauty is a universal phenomenon and debate over what constitutes beauty particularly beauty to human body, has raged since philosophy began. The beauty of individual features depends on “ideal” proportions, and it is suggested that expressing beauty in terms of geometry is possible. Assessment of some used parameters in facial surgeries and harmony of various facial features are essential to surgeon, who requires facial analysis. One of these parameters, is nasolabial angle, in patients undergoing rhinoplasty. This study based on theoretical definitions of beauty and proportions performed the search for the application of this numerical proportions in modern cosmetic surgery.

**METHODS:**

Twenty-three samples [16 (69.5%) female and 7 (30.5%)] male] were enrolled from patients who underwent rhinoplasty, by a single surgeon. The nasolabial angle was measured in these patients from their lateral profile photographs with adobe Photoshop, before and after surgery.

**RESULTS:**

Ideal post-operative angle was 111.54±26.5 degrees from this study and 18.8^◦^ increase in male and 14.68^◦^ increase in female were seen. There was no significant difference between men and women.

**CONCLUSION:**

Our results showed that an ideal proportion can be very useful and practical to assess patient’s preoperative expectations and to evaluate the results after surgery and satisfaction of cosmetic surgery process.

## INTRODUCTION

Esthetic is taken from the Greek word (Aisthanesthai), which means sensory perception.^1^ For Plato, something of our symmetry is included in what he means by beauty, and the long mathematical approach to symmetry starts with the Timaeus.^2^ Evidence from historical texts and art dating back to the Renaissance period show that appreciation of ideal facial proportions has persisted for ages.^3^ It was hypothesized that values of certain measured proportions in beautiful faces are likely to approximate the divine proportion.^4^ The rule of golden proportions has been proposed in an attempt to define anatomical beauty.^5^ A new challenge to face recognition is facial plastic surgery alters struggle to identify a person face after surgery.^6^

There is historical evidence for cosmetic surgery in ancient times.^7^ Cosmetic surgery is increasingly popular, globally.^8^ In contemporary society, the media are largely responsible for providing universal Yardsticks.^9^ Reports and recent comments suggest that beauty has become one of the main Iranians concerns. In one of the English-language sites, the report quoted the world Health Organization (WHO) called Iran as the world capital cosmetic surgery.^10^ Despite its subjective natures (beauty), we can attempt to define, measure and explain the captivating phenomenon of beauty by describing it numerically and geometrically.^11^

The number of people undergoing these plastic surgeries is increasing every day.^6^ A satisfactory cosmetic results and optimal healing is the aim of aesthetic surgery.^9^ It is essential therefore, to be able to assess the possible satisfaction that can be expected after an aesthetic surgery procedure and to determine the beauty of the final results as precisely as possible.^9^ All facial parts are of absolute importance for the perception of facial beauty. However, the nose has a special importance because it occupies the central position in the face.^12^ One of the most important parameters in the nose to measure, is tip rotation. 

An arbitrary range of 90 to 115 degrees for the nasolabial angle (in connection with the nasal tip rotation) is common.^12^ By measuring this parameter, the far and near obtained number in various researches from the ideal proportion can be identified, and the role of this parameter in assessing postoperative can be assessed. Assessment of some used parameters in facial surgeries and harmony of various facial features are essential to surgeon, who requires facial analysis. One of these parameters, is nasolabial angle, in patients undergoing rhinoplasty. This study based on theoretical definitions of beauty and proportions performed the search for the application of this numerical proportions in modern cosmetic surgery.

## MATERIALS AND METHODS

This study assessed the historical context and the origin of aesthetic beauty and numerical proportions philosophically, and after the history of cosmetic surgery, evaluated the use of the parameters of these proportions. In contemporary cosmetic surgery, a sample of 23 individuals who underwent rhinoplasty in a beauty clinic were enrolled and the angle between the lip and nose in these patients before and after surgery was determined to achieve the ideal angle. Finally, the results of this study were compared with previous researches to express application of the proportionality in modern surgery.

**Fig. 1:**
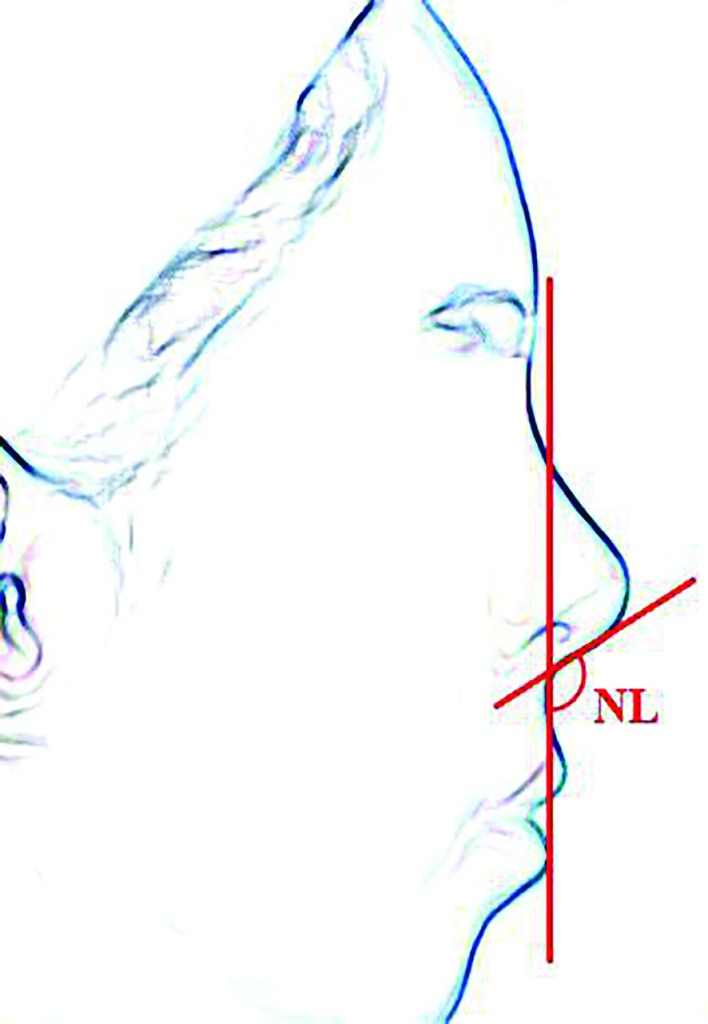
The most important criteria is tip rotation that is determined on angle base between the nose and lip

The present study was a retrospective approach that put the overview origin of these proportions, and used nasolabial angle which is widely used in proportions face surgery. Nasolabial angle is located between clomella and upper lip, while the angle measure is with less error. To have less error, the environmental variables and patients were selected from a single surgeon in one clinic. The samples were 23 patients, 7 men and 16 women with images of similar quality and position (lateral and forward head, sitting on a chair) that were similar in age class. Geometrical standards were of particular importance for aesthetic (Figure 1).

Rhinoplasty establishes a pleasant connection between nose and other facial components, in addition to their proportions in the nose. One of the most important criteria is tip rotation, that this amount is determined on angle base between the nose and lip. This angle is between the two lines as follows: (1) The line passes from front and back of nostril, (2) Vertical line to the horizontal plane when patient back forward, (3) We can easily measure mentioned angle with our available software, and (4) To take effective steps the more accurate way of doing rhinoplasty.

## RESULTS

Obtained results from this study indicated the ideal angle was about 111.54±26.5^◦^ after surgery. Average change of this angle in men had 18.8^◦^ increase and in women 14.68^◦^ increase. The average postoperative angle was significant sexual difference, despite the variety preoperative angle between male and female patients. Preoperative angle in men showed a more limited range, and obtained about 90-100^◦^, but in women were a wider range, and showed 87-110^◦^. 

After surgery, nasolabial angle was 106-117 in men and 106-114 in women, that indicated fixed numerical range of the procedure which can be considered an ideal range in patients undergoing rhinoplasty to evaluate the postoperative results. Results in Table 1 showed that maximum change was indicative of 24 degrees and minimum change was 12 degrees representing the average change had 18.8 degrees in male patients. Results in Table 2 showed that maximum change was 23 degrees, one was unchanged. The obtained results represented the average change had 14.68 degrees in female patients. Results from Table 3 were indicative of more changes in the postoperative angle of men. Range of preoperative angle in men was 90-100 degrees and in women was about 87-110 degrees. Average preoperative was 92.4 in men and 97.12 in women. Postoperative angle was 111.54±26.5 in both of them. Thus, as can be seen, changes of this angle was more in men because had they less preoperative angle.

**Table 1:** Pre- and post-operative nasolabial angle and its changes in men

**No**	**Preoperative angle **	**Postoperative **	**The change**
1 2 3 4 5 6 7	90^◦^ 90^◦^ 90^◦^ 90^◦^ 93^◦^ 94^◦^ 100^◦^	114^◦^ 112^◦^ 110^◦^ 107^◦^ 117^◦^ 106^◦^ 113^◦^	24^◦^ 22^◦^ 20^◦^ 17^◦^ 24^◦^ 12^◦^ 13^◦^

**Table 2:** Pre- and post-operative angle and its changes in woman

**No**	**Preoperative angle **	**Postoperative **	**The change**
1 2 3 4 5 6 7 8 9 10 11 12 13 14 15 16	87^◦^ 90^◦^ 90^◦^ 90^◦^ 90^◦^ 93^◦^ 95^◦^ 96^◦^ 98^◦^ 98^◦^ 100^◦^ 100^◦^ 102^◦^ 103^◦^ 110^◦^ 112^◦^	110^◦^ 106^◦^ 109^◦^ 112^◦^ 113^◦^ 110^◦^ 107^◦^ 113^◦^ 114^◦^ 115^◦^ 115^◦^ 106^◦^ 115^◦^ 115^◦^ 110^◦^ 121^◦^	23^◦^ 16^◦^ 19^◦^ 22^◦^ 23^◦^ 20^◦^ 15^◦^ 11^◦^ 15^◦^ 16^◦^ 15^◦^ 15^◦^ 4^◦^ 12^◦^ Fix 9^◦^

**Table 3:** Pre- and post-operative nasolabial angle in woman and men and change extent

**Sex**	**Pre-operative angle extent**	**Post-operative angle extent**	**Average pre-operative**	**Average post- operative**	**Average of change**	**Average of whole angle**
Men Women	90-100 88-110	106-117 106-114	92.4 97.12	111.28 111.81	18.8 14.68	111.5±26.5 111.5±26.5

## DISCUSSION

In a study that was conducted in 37 patients in 2008, prospective analysis of 37 patient submitted to rhinoplasty, were 13 (36%) men and 24 (64%) women. The nasolabial angle was measured and compared, before and after surgery, in lateral profile pictures. An average increase of 8.6^◦^ in the nasolabial angle was observed.^13^ In Turkish people (56 males and 59 females), this angle was achieved without surgery about 98.91±2.32^◦^ in female.^14^

Among 102 adults (41 man and 61 women) in the south Indian population, the obtained ideal angle was about 99.76^◦^. No difference between both sexes was observed.^15^ In 2006 lateral photographs were taken of 100 volunteers (60 women and 40 men), the nasolabial angle for females was about 102.22^◦^ and for males was 98.83^◦^.^16^ In Bangladeshi among both male and female population in absence of surgery, the mean value of nasolabial angle was 91.28^◦^±12.98^◦^ in males and 91.92^◦^±8.90^◦^ in females. The difference was not statistically different.^17^

Among 20 patients (18 females and 2 males) pre-operative nasolabial angle was 99±8.82^◦^ and post-operative nasolabial angle was 104.5±8.25^◦^.^18^ Another study was done among 45 patients (24 females and 21 males) without any significant difference between men and women, while the mean nasolabial angle was 96.1±9.7.^19^ Since ancient times, the supporters of beauty as an objective and measurable property attempted to state ideal proportions, or beauty canons, for the human body and its part,^20^ and the face was considered beautiful with harmonious features if the individual components were proportional.^21^

A new challenge to face recognition is facial plastic surgery.^6^ While Iran ranks first in cosmetic surgeries and has been called the capital of world’s rhinoplasty,^7^^,^^10^ the facial proportions are considered essential to help the surgeon who requires facial analysis in the diagnosis and treatment plannings.^22^ Therefore, the relevant parameters can be a way for satisfaction after surgery and a satisfactory cosmetic result and optimal healing is the aim of every aesthetic surgery.^9^ Generally, philosophical ideas about beauty and art has existed since Plato onward across the west.^1^

The sense of aesthetic was employed first by Alexander Gottleb Baumgarten in a book as Latin name was Aesthetica (Same). Body measurement were used by the old Egyptians to execute their famous sculptures and painting facial measurements as first performed by the Greeks for measurements of total body and for the same purpose.^9^ History of human life has been full of worship or as beautiful creatures and this is not to say that, what kind of beauty was worshiped while it is without change.^23^ Rules defining the relationships between various face and body features were more clearly formulated by scholars and artists of the Renaissance based on classical Greek canons before.^9^

From the era of the ancient Greeks, through to the Renaissance, and the present day, mathematicians, scientists, architects, artists, and cosmetic surgeons have been intrigued by the ubiquitous nature of the divine proportion and its correlation with aesthetics.^11^ In Plato’s classic aesthetics, sensible beauty was shadows, effects, or idea from conceptional beauty.^1^ Debate over what constitutes beauty of the human body, has raged since philosophy began.^9^ The old age beauty lies in the eye of the beholder.^4^^,^^24^^,^^25^ They stipulated that the individual judgements were paramount and needed to be regarded since the assessment of facial attractiveness is very complex.^25^

Aristotle put just once a benchmark offering a beautiful tragedy that should not be neither too long and not to have enough memory to record, nor to be too short.^26^ This piece shows that beauty can be defined using the length (more volume) and ratio (same). It appears that youth and symmetry are the most highly prized attributes of beauty.^9^ Beauty and facial attractiveness are easy to identify but difficult to quantify.^11^ Modern life style, constantly influenced by media exposure of universal beauty standard, gives aesthetic values a pivotal role in social life.^27^

Since ancient times, the supporters of beauty as an objective and measurable property attempted to state ideal proportions, or beauty canons for the human body and its parts.^28^ The harmonic body shop as perceived by the human eye is a result of a series of definite numeric relationships between the sizes and positions of various segments of the body.^24^ Furthermore, geometric patterns and the numbers associated with them gave symbolic role to this system with holy concepts that permanent arch type or as jung, draw primordial role in the proper pattern with the artistic language, until being standard for human.^29^

The golden ratio also known as the divine proportion, is considered by many to be the key to the secret of aesthetics, attraction and human beauty.^11^^,^^21^ Renaissance artist, as Leonardo da vinci, Leon Battista Alberti, Alberecht Duerer and Piero della Francesca, reformulated and documented the classic canons, that have been used for centuries in art by sculptors, painters, and are a rough working guide for plastic surgeons.^28^ More precisely, aesthetic judgments can be considered a subset of evaluative judgments too.^9^ Although certain characteristics of human faces are broadly considered more attractive (e.g., symmetry, averageness), people also routinely disagree with each other on the relative attractiveness of faces.^30^

The first record history of plastic surgery in the world is related to 600 BC, In Iran recorded history of plastic surgery goes back to 1000 years ago, the time of Ibn Sina introduced the first repair of the tendon. According to recent statistics by the American Society of Plastic Surgery in 2008, more than one million facial plastic surgeries were performed, with a growth of 162% in ten years.^28^ Some theorist believe that feminization of cosmetic surgery is likely to be short-lived historically. Statistics and tends also confirmed so that is indicated a gradual increase in men and non-whites happened.^7^

In our study, application of numerical proportions in contemporary cosmetic surgery was assessed. So one of the important parameters in pre- and post-rhinoplasty’s surgery assessment was selected. Nasolabial angle was selected for assessment of tip rotation as important factor for cosmetic surgeons, and the special and practical aims of this study were to gain an ideal proportion as a measures of assessment before surgery, for the patient expectation and after surgery for satisfaction from surgery procedure. Our results indicated a relatively fixed number as an ideal proportion of assessment for pre- and post-operative procedure.

The results were obtained in small samples of patients in a beauty clinic, so it is recommended to examine more patients and several beauty clinics in next study, to generalize the value and decrease the limitation of this study. The perspective, preoperative angle in this study was closest to another study.^17^ The average ideal angle in this study regarding the preoperative angle in men (90-100^◦^) was 92.4^◦^, that was closest to Garo’s results without surgery (91.28^◦^ in men).^17^ Our findings were closest to Dua *et al.*’s study in 2010, (96.1±9.1).^19^ Preoperative angle in this study was closest to another study in 2014 (98.91±10^◦^) in women.^31^ Our study results about preoperative angle in women was also close to Dua *et al. *in 2010 (96.1±9.7^◦^).^19^

The present study on post-operative angle in male and female patients was closest to Pasinato *et al.*’s study in 2007 (107.6±7.5^◦^)^13^ and another study in 2013 (females 107.57^◦^ and male: 105.2^◦^).^25^ Our finding was also close to Meruane *et al.* in 2016 (104.5±8.25).^18^ Post-operative angle obtained from this study was 111.54±26.5^◦^, that was far from another study (93.4-98.5^◦^ in male and 95.5±100^◦^ in female).^14^ This study was also far from Kommi *et al.*’s study in 2015 (99.76±15.35),^15^ but was close to across the whole, between two obtained proportion. The present study was close to Aghili *et al.* in 2016 (102±10.22) across the whole, this ideal proportion had achieved without surgery in usual persons.^32^

In European white skin people, the ideal angle without surgery was closest to the ideal proportion of our finding in patients without surgery. This is may be a marker of the desire in Iranians to westernize their ideal proportions and their desire to correct facial proportion according to a normal western face standard. Other limitations of our study were ignoring the ethnicity, race and genetic features and age. In Aghili *et al.*’s study in Iran,^32^ the age factor was also taken in to account. However, in our study, it reduced with age increase. 

The used techniques were different in various researches. Some of them had used radiographic methods, some direct measurement such as anthropometric methods and some methods were assessment of pictures and photos. It can be another limitation of our study which can be effective in the evaluation process. Finally, our results showed that an ideal proportion can be very useful and practical to assess patient’s preoperative expectations and to evaluate the results after surgery and satisfaction of cosmetic surgery process, but to reach the best results and make the right decisions, ethnicity and genetic and age factors and other nose proportions with other face members should be taken into account until the obtained proportion is in accordance with the figure that is expected of a specific ethnicity, to look more beautiful before surgery.

## CONFLICT OF INTEREST

The authors declare no conflict of interest.
